# Preparation and characterization of a certified reference material of toxic elements in cannabis leaves

**DOI:** 10.1007/s00216-025-05809-z

**Published:** 2025-03-12

**Authors:** Adriana Rodriguez, Cristhian Paredes, Elianna Castillo

**Affiliations:** 1 Grupo de Investigación en Metrología Química y Bioanálisis, Instituto Nacional de Metrología de Colombia, Ak. 50 No. 26-55 Int. 2, Bogotá, 111321 Colombia; 2 Departamento de Química, Facultad de Ciencias, Grupo de Estudios para la Remediación y Mitigación de Impactos Negativos al Ambiente, Universidad Nacional de Colombia, Ak. 30 No. 45-3, Bogotá, 111321 Colombia

**Keywords:** Cannabis, Certified reference material, Toxic elements, Metrological traceability, Human health

## Abstract

The quantification of harmful elements in cannabis is a relevant analytical task that requires metrological tools to ensure the reliability of the measurement results. This work reports the preparation and characterization of a certified reference material (CRM) for toxic elements—arsenic (As), cadmium (Cd), and lead (Pb)—in pulverized, lyophilized cannabis leaf tissue. To prepare the CRM INM-040-1, a portion of dried ground cannabis vegetal material was spiked with the elements at mass fraction values close to the levels of interest in relevant regulations for this kind of matrix: 0.34 mg/kg for arsenic, 0.34 mg/kg for cadmium, and 0.66 mg/kg for lead. The elements were quantified by inductively coupled plasma mass spectrometry (ICP-MS) in combination with graphite furnace atomic absorption spectroscopy (GF-AAS, for cadmium and lead) or hydride generation atomic absorption spectroscopy (HG-AAS, for arsenic). The analytical calibration was done by gravimetric standard addition for ICP-MS and GF-AAS, while bracketing calibration was used for HG-AAS. Furthermore, internal standard correction was used during ICP-MS measurements. The analytical methods were validated to demonstrate their fitness for purpose. The preparation variables of the CRM (particle size, drying treatment, and spiking conditions) were studied to improve the homogeneity of the CRM. The mass fraction of the toxic elements was certified with relative standard uncertainties ranging from 4.2 to 6.9%. The uncertainty contributions considered were the elements’ mass fraction measurements, the between-methods bias, the (in)homogeneity of the production batch, and the (in)stability under transport and storage conditions. This new CRM constitutes a useful tool for the laboratories assessing the harmlessness of cannabis materials, promoting humans’ safety and regulatory compliance within the medicinal cannabis industry.

**Graphical Abstract:**

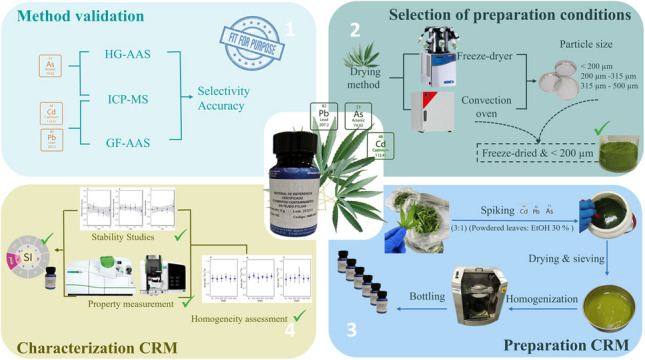

**Supplementary Information:**

The online version contains supplementary material available at 10.1007/s00216-025-05809-z.

## Introduction

The acknowledgment of diverse therapeutic benefits of cannabis-derived products has fostered the development of an industry that contributes from the bioeconomy to create innovative healthcare solutions. The cannabis plant has positioned as a renowned feedstock in numerous medicinal and industrial applications in the framework of reciprocal relationships among scientific, cultural, economic, and legislative advances [1, 2]. The growing importance of cannabis has prompted regulatory agencies and international organizations related to the pharmaceutical and food sectors to set guidelines that establish quality parameters for the cannabis plant and cannabis-derived products [3–5]. The quality parameters consider the content of desirable components, namely cannabinoids and terpenes, responsible for the beneficial characteristics of the cannabis plant, and the absence of significant amounts of undesirable contaminants that pose a risk to human health: mycotoxins, pathogenic microorganisms, pesticide residues, and toxic elements [5].

The innocuity of cannabis concerning the absence of toxic elements is of special interest because the cannabis plant tolerates growing media with high concentration of cadmium, chromium, nickel, and lead, among other toxic elements [5, 6] and has the potential to bioaccumulate them to a significant extent [7, 8]. This characteristic makes the cannabis plant an attractive phytoremediator for polluted areas [7], but also implies that the vegetal material should be tested for harmful elements before processing. Ensuring the quality of the measurement results from testing laboratories serving the cannabis value chain is essential for the safe and effective use of cannabis as a pharmaceutical resource.

The world’s National Metrology Institutes (NMI) develop tools and strategies to support the quality of the measurements required to improve peoples’ well-being, promote the protection of the environment, and overcome potential barriers to commerce [9]. An important metrological tool in chemistry and biology is the certified reference material (CRM). A CRM is a material sufficiently homogeneous and stable regarding specified properties which have been determined using valid procedures [10]. CRMs are used in the development and validation of measurement methods and to perform quality control during routine analysis. Because of the early stage of the cannabis industry, few CRMs have been developed to date in this matrix. Notably, the National Research Council from Canada (NRC) developed the CRM HEMP-1 which is intended for the method development and validation and the quality control of the analysis of cannabinoids in cannabis and hemp [11]. Additionally, the National Institute of Standards and Technology (NIST) from the USA developed Reference Material 8210 which provides non-certified values for cannabinoids and toxic elements in dried ground hemp [12].

In Colombia, the Instituto Nacional de Metrología (INM(CO)) is increasing its commitment to support the quality of the measurements in the cannabis value chain, as the cannabis might transform into the next commodity that will help diversify the Colombian export market [13]. In this context, the objective of this work was to prepare and characterize a CRM for toxic elements in cannabis leaf tissue. The CRM INM-040-1 is intended as a metrological tool for the development and validation of analytical methods and the quality control of measurements of toxic elements in hemp and cannabis. The material was characterized according to the requirements set by the ISO Standard 17034 [14] using the combination of two independent methods of demonstrable accuracy to measure the elements’ mass fraction: cadmium and lead were determined by inductively coupled plasma mass spectrometry (ICP-MS) and graphite furnace atomic absorption spectroscopy (GFAAS). In the case of arsenic, it was determined using both ICP-MS and hydride generation atomic absorption spectroscopy (HG-AAS). Additionally, the CRM’s homogeneity and its stability under simulated transport conditions and recommended storage conditions were assessed with ICP-MS using established experimental designs [15]. The fitness for purpose of the methods was confirmed by validation of the analytical methodologies using CRMs of similar vegetal matrices.

## Materials and methods

### Chemical reagents and CRMs

The metrological traceability in analytical calibration of measurement methods was established using the 3100 series Standard Reference Materials (SRM®) from NIST (USA): SRM 3128 lead (Pb), SRM 3108 cadmium (Cd), and SRM 3103a arsenic (As). The method validation was made using CRMs with a similar matrix to that of the cannabis leaves: SRM 3254 (tea leaves), SRM 1515 (apple leaves), SRM 1573a (tomato leaves), SRM 1575a (pine needles), and SRM 3232 (powdered algae), all from NIST. The ICP-MS measurements used internal standard solutions of thallium (TraceCERT, Sigma-Aldrich, USA), rhodium, indium, and germanium (Supelco, Merck, USA). Ultrapure water with conductivity of 0.058 µS cm^−1^ was obtained using a PURELAB Flex purification system (Elga LabWater, High Wycombe, UK). Ethanol (absolute, for analysis, Supelco, Merck, USA) was used during spiking experiments of the CRM. Nitric acid and hydrogen peroxide (Suprapur, Merck, Germany) were used for the microwave-assisted digestion of samples. The nitric acid was doubly subdistilled (DST-1000, Savillex, USA) to further reduce the content of possible impurities. For arsenic analysis by HG-AAS, magnesium nitrate hexahydrate (Panreac, Spain) and magnesium oxide (reactive grade, Merck, Germany) were used for sample ashing. Potassium iodide (Panreac, Spain) and l(+)-ascorbic acid (EMSURE® Merck, Germany) were employed in the pre-reduction step. Finally, sodium hydroxide (EMSURE® Merck, Germany) and sodium borohydride (ReagentPlus® 99% Sigma-Aldrich, USA) were used in the reduction step within the hydride generator.

### Pilot material processing studies

A pilot batch of cannabis vegetal material was processed to preliminarily assess the preparation conditions that enhance the homogeneity of the CRM. The drying method (oven drying and freeze drying) and the particle size after grinding were studied. For this stage, 200 g of cannabis leaves was collected from an artisanal crop (San Antonio del Tequendama, Cundinamarca, Colombia). For oven drying, the cannabis leaves were heated to 80 °C in a convection oven (ED 115-UL, Binder, Alemania) for 24 h, while for freeze drying, the leaves were frozen at −20 °C and then lyophilized at approximately 3 kPa (FreeZone, Labconco, USA) for 120 h. After drying, the leaves were processed in a grinder until a fine powder was obtained. Three particle size fractions (<200 µm, 200–315 µm, and 315–500 µm) were obtained using stainless steel sieves (FRITSCH, Spain). The combination of two drying procedures and three particle sizes yielded six treatments that were evaluated in terms of the relative standard deviation (RSD) of the measurement results of the element’s mass fraction (five measurements of independent subsamples).

### Preparation of the CRM, spiking experiments, and bottling

After the preliminary preparation studies, 1.6 kg of cannabis vegetal material was obtained from a licensed cultivation company (Llanos Oil, Fusagasugá, Colombia). The leaves were freeze dried, grinded, and sieved, and the size fraction under 200 µm retained. A preliminary quantification of arsenic, cadmium, and lead performed via ICP-MS yielded no quantifiable amounts of the elements; therefore, spiking was necessary to achieve the target mass fraction.

Spiking trials were conducted with 40 g portions of the cannabis leaf powder. Water-ethanol was proposed as the spiking solvent due to the high content of waxes in the cannabis leaves. The mass fraction of ethanol in the water-ethanol mixture (10% and 30%) and the influence of an acidic medium were evaluated (a total of four treatments). The slurry was prepared at a solid-to-spiking solution ratio of 1:3 and thoroughly stirred for 30 min. The four different pastes were freeze-dried, grinded, and sieved. The spiking treatments were compared again in terms of the RSD of five independent measurements of arsenic, cadmium, and lead in separate subsamples. The selected spiking condition (30% ethanol in water without acidification) was applied to 380 g of processed cannabis leaf powder, obtained after drying, grinding, and sieving. This portion was incorporated into 1140.1 g of an aqueous solution of arsenic, cadmium, and lead at mass fractions 0.114 mg kg⁻^1^, 0.115 mg kg⁻^1^, and 0.214 mg kg⁻^1^, respectively, The slurry was shaken for 30 min, followed by freeze-drying, grinding, and sieving. The spiked dry material was thoroughly homogenized for 30 min in a gyroscopic mixer (GyroMixer, Fluid Management) and aliquoted in amber glass bottles with 5 g of the CRM. A total of 48 bottles were obtained and stored at room temperature.

### Element quantification

For ICP-MS and GF-AAS analysis, sample portions of 0.5 g were weighed directly into modified polytetrafluoroethylene (PTFE-TFM) digestion vessels, and 2 mL of hydrogen peroxide and 5 mL of sub-distilled nitric acid were added. The vessels were tightly closed and microwave-digested under constant temperature monitoring (Multiwave 5000, Anton Paar, Austria) using the temperature program presented in Table 1. After digestion, the samples were cooled to room temperature, degassed, and quantitatively transferred to polypropylene centrifuge tubes for gravimetric dilution after the addition of internal standard. All the gravimetric measurements were performed on a calibrated XPE 504 balance (Mettler Toledo, USA).

**Table 1:** Temperature program for the microwave-assisted digestion of the cannabis samples

Step	Final temperature, °C	Ramp time, min	Hold time, min
1	145	15	20
2	190	10	15

ICP-MS measurements of arsenic, cadmium, and lead were performed using a single-quadrupole instrument (NexION 300D, PerkinElmer, USA). The plasma was generated using argon grade 5.0 with a radiofrequency power of 1300 W, plasma gas flow of 13.1 L min⁻^1^, auxiliary gas flow of 1.35 L min⁻^1^, and nebulizer gas flow ranging from 0.56 to 0.85 L min⁻^1^. A quartz cyclonic spray chamber, Meinhard nebulizer, and nickel cone system were employed. The torch position, nebulizer gas flow rate, and deflector voltages were optimized daily to maximize analyte signals and reduce interferences from polyatomic ions, doubly charged ions, and oxide species. Calibration was performed using gravimetric standard addition calibration with internal standard correction.

GF-AAS measurements (PINNACLE 900T equipped with an AS 900 autosampler, PerkinElmer, USA) were made for cadmium and lead using the temperature programs and conditions shown in Table 2. The matrix modifier for cadmium was 30 µg of palladium, while lead used a combination of 30 µg of palladium and 3 µg of magnesium. Calibration was performed using gravimetric standard addition calibration.

**Table 2:** Temperature programs in GF-AAS for the measurement of cadmium and lead

Element	Step	Temp, °C	Ramp time, s	Hold time, s	Flow internal gas (mL/min)
Cd (228.80 nm) slit width: 0.7 nm	Evaporation	110	1	30	250
	Evaporation	130	15	30	250
	Calcination	750	10	20	250
	Atomization	1500	0	3	0
	Cleaning	2400	1	3	250
Pb (283.31 nm) slit width: 0.7 nm	Evaporation	110	1	40	250
	Evaporation	160	20	35	250
	Calcination	700	10	20	250
	Atomization	1900	0	5	0
	Cleaning	2400	1	5	250

HG-AAS measurements (PINNACLE 900T coupled with FIAS 400, PerkinElmer, USA) were used for arsenic determination. The sample preparation procedure described in [16] was used. In summary, sample portions of 0.5 g were weighed in porcelain crucibles and added with 2 mL of an aqueous suspension of 20% magnesium nitrate (MgNO₃) and 2% magnesium oxide (MgO) and 5 mL of a 1:1 HNO_3_:H₂O solution. The mixture was homogenized before evaporating the solvent avoiding ebullition. A calcination temperature program (150 °C for 1 h, 200 °C for 30 min, 250 °C for 1 h, 300 °C for 3 h, 350 °C for 30 min, and 450 °C for 12 h) was performed in a muffle furnace (FHX-05, Daihan Scientific, Korea). The ashes were resuspended in 5 mL of a 1:1 HNO_3_:H₂O solution, evaporated, and calcinated again using a shorter temperature program (300 °C for 3 h, 350 °C for 30 min, and 450 °C for 12 h). After the second calcination, white homogeneous ashes were obtained and dissolved in 5 mL of a 1:1 HCl:H₂O mixture and brought to the final mass with ultrapure water. Before HG-AAS analysis, the extracts were pre-reducted by mixing 50% reconstituted sample extract, 10% concentrated hydrochloric acid, and 25% of a potassium iodide and ascorbic acid solution (10%), with a reaction time of 45 min. The reduction step involved the use of a 0.2% sodium borohydride solution in 0.05% sodium hydroxide as the reducing agent and a 5% hydrochloric acid solution as the carrier. The instrumental conditions were as follows: the wavelength was set to 193.7 nm with deuterium background correction. The argon flow rate was maintained between 40 and 50 mL min⁻^1^, and the electrothermal chamber temperature was set to 960 °C. A 500 µL sample injection loop was used for sample introduction.

All analytical methods were validated in terms of their instrumental linear range, accuracy (precision and trueness), and selectivity. The instrumental linear range was evaluated in ICP-MS in the interval 0.5 to 100 µg kg⁻^1^ for the three elements. In the case of GF-AAS, cadmium was evaluated from 0.5 to 10 µg kg⁻^1^, while lead was evaluated from 3.0 to 33 µg kg⁻^1^. For HG-AAS, arsenic was evaluated from 1 to 15 µg kg⁻^1^. The lack of fit ANOVA was used to identify deviations from linearity in the assessed ranges [17]. The precision of the methods is expressed in terms of the relative standard deviation from five independent measurements of each of the CRMs. The trueness is assessed in terms of the relative bias against the certified values of the CRMs. The statistical significance of the bias uses the normalized error (*E*_n_) as defined in Eq. 1 [18]. Normalized error values smaller than 1 imply that the measurement bias is not statistically significant. Furthermore, as the CRMs contain several elements other than those of interest, a non-statistically significant bias serves also as proof of the selectivity of the methods.

1 $${E}_{\text{n}}=\frac{\left|{x}_{\text{CRM}}-\overline{{x}_{i}}\right|}{2\sqrt{{\left({u}_{\text{CRM}}\right)}^{2}+{\left({u}_{{x}_{i}}\right)}^{2}}}$$

where ${x}_{\text{CRM}}$ is the value of reference, $\overline{{x }_{i}}$ represents the measured value, and ${u}_{\text{CRM}}$ and ${u}_{xi}$ are the uncertainties of the CRM and the measured value, respectively.

The CRMs used for the validation of the methods were also used as quality controls during the property value measurements of the cannabis CRM.

## Results and discussion

### Fitness for purpose of the analytical measurement methods

The standard addition calibration model selected for the ICP-MS method uses a first-degree linear equation that requires all measurements of samples and spiked solutions to fall in the linear range of the instrumental response. This requirement does not hold for standard addition approaches that do not rely on first-degree linear models [25]. The linearity was tested by lack of fit ANOVA, and the elements’ mass fraction working ranges were trimmed until the lack of fit test succeeded. The instrumental response of the ICP-MS instrument varied linearly across the evaluated mass fraction intervals for most cases. The accepted linear ranges and the resulting *p*-values of the lack of fit ANOVA tests for the ICP-MS instrumental responses are presented in Table S1 of the supplementary information. A *p*-value of the lack of fit ANOVA larger than the significance of the test (typically 0.05) implies that a higher order polynomial model is not significantly better than the simple linear model in describing the relation between element’s mass fraction and instrumental response [17]. Therefore, the analyte mass fraction in the standard spiked sample solutions should not exceed the upper limit of the linear ranges presented in Table S1. The same analysis was made to determine the working range of both the GF-AAS and HG-AAS methods. Table S2 of the supplementary information presents the linear range results obtained for the GF-AAS and HG-AAS methods for cadmium, lead, and arsenic.

The accuracy of the ICP-MS and GF-AAS methods was tested using three vegetal CRMs with varying mass fraction of the analytes, while HG-AAS was assessed with two vegetable CRMs, as SRM 1575a has an arsenic mass fraction lower than the method’s limit of quantification. The results are summarized in Table 3. Precision under repeatability conditions is expressed as the RSD of the results from five independent measurements performed on separate subsamples. The larger dispersion (5.3% RSD) was obtained for lead using ICP-MS at the mass fraction of 0.167 mg/kg (SRM 1575a, pine needles). Other repeatability RSDs range from 0.8 to 4.4%. The obtained values are significantly smaller than the limits stated in the “Standard Method for the Determination of Heavy Metals in a Variety of Cannabis and Cannabis-Derived Products, 2021.03” [4], which sets a maximal RSD value of 11% for elements in a mass fraction between 0.1 and 1 mg kg⁻^1^. Regarding the trueness of the methods, the measurement methods used do not have significant bias, and the maximal relative error for the measurements was 5.4% [4].

**Table 3:** Accuracy testing for arsenic, cadmium, and lead at three mass fraction levels, using ICP-MS and GF-AAS and HG-AAS

Analytical technique	Element	CRM	Mass fraction^a^, mg kg^−1^	RSD, %	Certified value^b^, mg kg^−1^	Relative error, %
ICP-MS	As	Pine needles SRM 1575a	0.041 ± 0.002	2.6	(0.039 ± 0.002)^c^	5.1
		Green tea leaves SRM 3254	0.153 ± 0.007	4.0	0.150 ± 0.011	2.0
		Kelp powder SRM 3232	37.0 ± 3.5	3.4	38.3 ± 1.3	−3.4
	Cd	Green tea leaves SRM 3254	0.039 ± 0.002	2.3	0.037 ± 0.002	5.4
		Pine needles SRM 1575a	0.231 ± 0.006	0.8	0.233 ± 0.004	−0.9
		Tomato leaves 1573a	1.483 ± 0.027	0.9	1.517 ± 0.027	−2.2
	Pb	Pine needles SRM 1575a	0.168 ± 0.013	5.3	(0.167 ± 0.015)^c^	0.6
		Apple leaves SRM 1515	0.465 ± 0.030	3.5	0.470 ± 0.024	−1.1
		Kelp powder SRM 3232	1.062 ± 0.026	1.9	1.032 ± 0.039	2.9
GF-AAS	Cd	Pine needles SRM 1575a	0.236 ± 0.010	1.9	0.233 ± 0.004	1.3
		Kelp powder SRM 3232	0.441 ± 0.014	3.4	0.426 ± 0.0084	3.5
		Tomato leaves SRM 1573a	1.548 ± 0.109	2.6	1.517 ± 0.027	2.0
	Pb	Apple leaves SRM 1515	0.484 ± 0.020	2.1	0.470 ± 0.024	3.0
		Kelp powder SRM 3232	1.029 ± 0.135	2.4	1.032 ± 0.039	−0.3
		Green tea leaves SRM 3254	1.72 ± 0.19	4.4	1.73 ± 0.19	−0.6
HG-AAS	As	Green tea leaves SRM 3254	0.149 ± 0.045	1.7	0.150 ± 0.011	−0.6
		Kelp powder SRM 3232	37.3 ± 0.1	2.4	38.3 ± 1.3	−2.6

^a^Expanded uncertainties with *k* = 1.97. ^b^Expanded uncertainties with *k* = 2. ^c^Non-certified value

The selectivity of a measurement method refers to its ability to unequivocally detect the analyte of interest in the presence of the matrix. Issues with selectivity in measurement methods can cause bias in the results [19]. In this study, the selectivity of the measurement methods was confirmed by the non-statistical significance of the measurement bias as assessed through the normalized error. The results shown in Table 3 indicate that the bias was not significative for any of the analytes in the mass fraction levels for all analytical techniques applying Eq. 1. These results confirm that the ICP-MS and GF-AAS methods with microwave-assisted digestion and HG-AAS with ashing mineralization are suitable for characterizing the CRM of toxic elements in cannabis.

### Pilot material processing studies: particle size and drying method

**Fig. 1:**
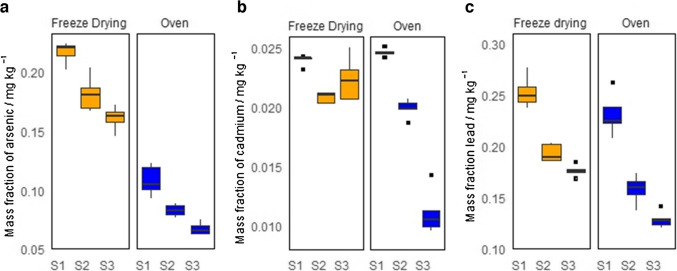
Measurement results for arsenic (**a**), cadmium (**b**), and lead (**c**) of the pilot cannabis material, processed using different drying methods and selecting different particle size ranges: smaller than 200 µm (S1), between 200 µm and 315 µm (S2), and between 315 µm and 500 µm (S3)

The pilot batch of the artisanal-grown cannabis material was processed with accordance to the six possible treatments that result from combining the two drying methods with the three particle size ranges. The mass fractions measured in five independent subsamples of each processed material are presented in the boxplots of Fig. 1. In general, greater variability is observed for the oven-dried material when compared with the freeze-dried cannabis. Furthermore, arsenic presents a drastic reduction when the cannabis material is dried in the convection oven. This variation can be attributed to the volatility of arsenic species at temperatures above 50 °C [20]. The dimethylarsinic acid ((CH₃)₂AsH) and trimethylarsine ((CH₃)₃As) are common species found in plants [21] and have boiling points of 36 °C and 52 °C, respectively [22]. This suggests that the freeze drying method should be preferred over the convection oven heating method. On the other hand, the mass fraction of elements increases systematically as the particle size decreases. This increase in the element’s mass fraction is accompanied by a reduction in the RSDs of the measurement results. As lower RSD is an indicative of a better sample homogeneity, the particle size fraction under 200 µm was selected for further preparations of the cannabis CRM.

### Preparation of the CRM candidate and spiking experiments

**Fig. 2:**
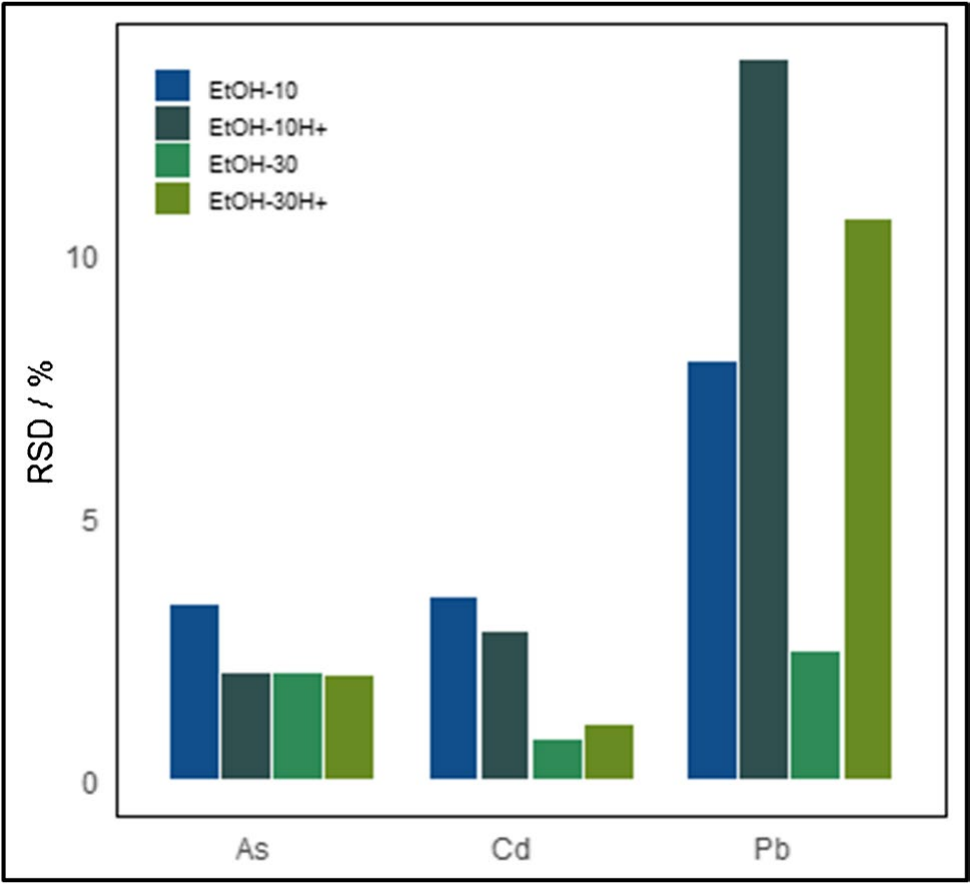
Relative standard deviation for cannabis leaves using different spiking solutions: EtOH-10: 10% ethanol solution; EtOH-10H+: 10% ethanol solution in acidic medium; EtOH-30: 30% ethanol solution; EtOH-30H+: 30% ethanol solution in acidic medium

The cannabis vegetal material obtained from a licensed producer was processed according to the method suggested by the pilot material processing studies: freeze drying, grinding, and particle size selection under 200 µm. After processing, the material did not contain any measurable amounts of arsenic, cadmium, nor lead. Therefore, it was deemed necessary to spike the cannabis material, as described in the “Preparation of the CRM, spiking experiments, and bottling” section. The cannabis leaves are hydrophobic due to a high content of waxes. To ensure that the spiking cocktail would intermingle with the vegetal material and wet the ground leaves at a considerable extent, ethanol was incorporated as an organic solvent in the fortification solutions. Two different volume fractions were tested: 10% and 30%. The effect of acidifying the spiking solution was also evaluated by incorporating 1% nitric acid in some of the solutions. The RSDs of the subsample measurements of the spiked treatments are presented in Fig. 2. The element lead presents the higher variation in the subsample measurements, with the best homogeneity obtained for the non-acidified spiking solution containing 30% ethanol. This spiking solution, coded EtOH-30, also gave the best results for cadmium element. The dispersion of arsenic subsample measurements did not vary significantly across the evaluated conditions. Accordingly, the remaining cannabis vegetal material was treated with the EtOH-30 spiking solution. After mixing thoroughly, the slurry was lyophilized and ground, and the particle size fraction under 200 µm retained, according to the conclusions drawn from the pilot material processing studies.

### CRM homogeneity assessment

**Fig. 3:**
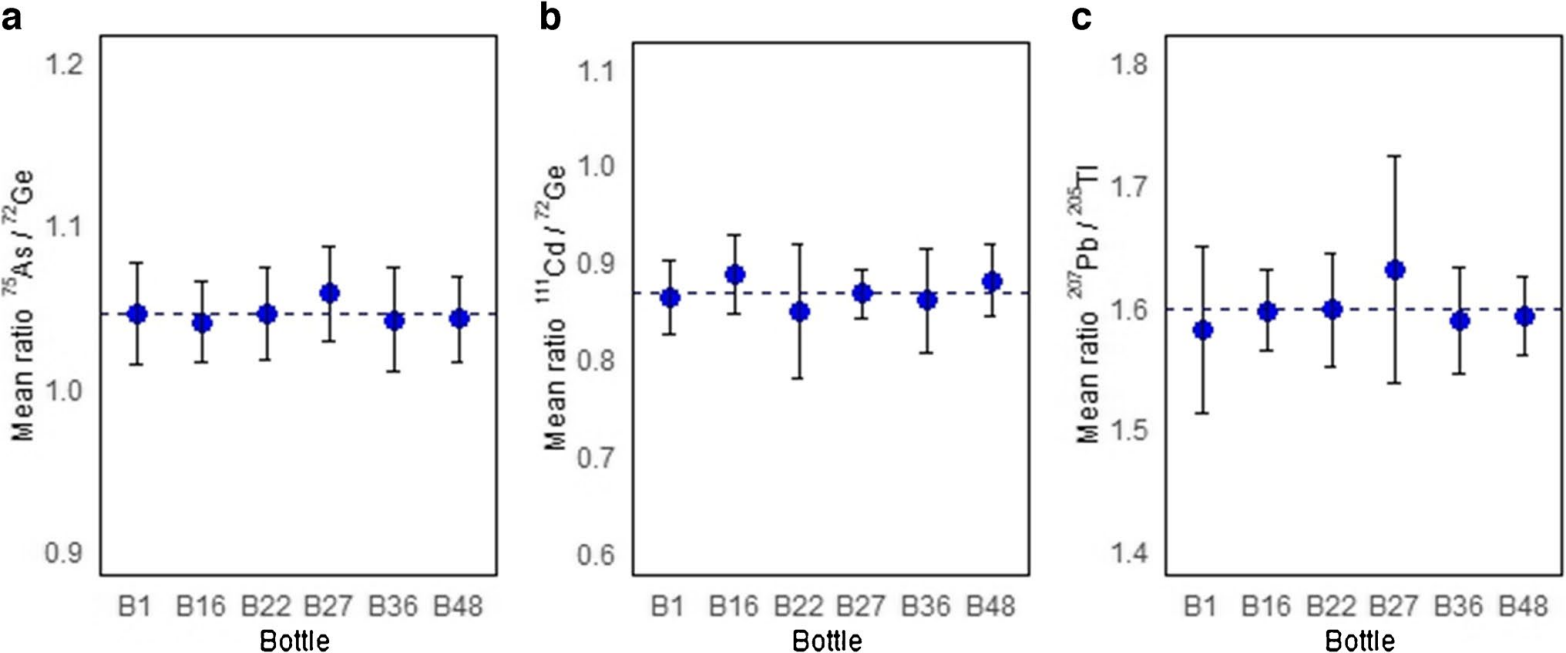
Homogeneity study for six randomly selected bottles of the production batch. The error bars indicate standard deviation of six subsamples mean results for arsenic (**a**), cadmium (**b**), and lead (**c**). The error bars indicate standard deviations of the subsample measurements per bottle

Six units from the CRM batch were selected using a stratified random sampling design that included intentionally the first and the last bottle. The homogeneity was assessed using a nested experimental design that allowed to determine the variation between bottles and within bottles: Six subsamples were taken per bottle, and each subsample was analyzed ten times under repeatability conditions. The data was processed in terms of the ratio of the analyte intensity to the internal standard intensity using ICP-MS. The recommendations of ISO Guide 35 [15] were followed for the estimation of uncertainty due to the heterogeneity of the CRM. The measurements were performed in random order. The absence of significative trends due to instrumental drift and the packaging order was verified before analyzing the data. Figure 3 presents the isotope ratio measurements for the bottles selected for the homogeneity experiment. The uncertainty due to homogeneity took into account the contributions from both between- and within-bottle evaluations using Eq. 2 [15].

2 $${u}_{\text{homo}}=\sqrt{{{(u)}^{2}}_{\text{between}}+ {{(u)}^{2}}_{\text{within}}}$$

where ${u}_{\text{between}}$ and ${u}_{\text{within}}$ are between- and within-bottle uncertainty in the stability study, respectively.

The decomposition of variances from experimental error, between-bottle variation, and within-bottle variations, as well as the calculation of each uncertainty, was carried out as described by Ellison [23]. The homogeneity uncertainties were 1.80% for arsenic, 4.89% for cadmium, and 2.22% for lead.

### Stability studies

**Fig. 4:**
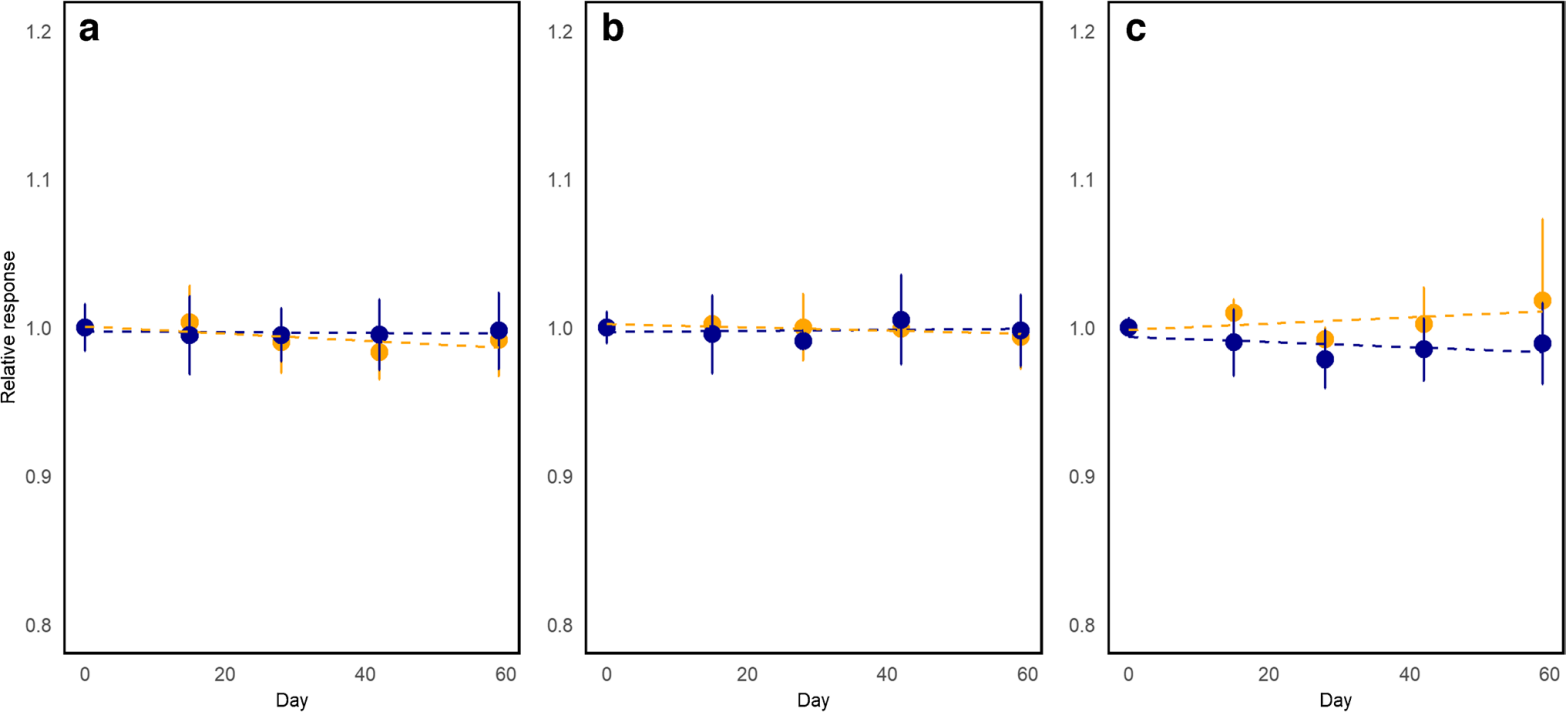
Isochronous short-term stability study of arsenic (**a**), cadmium (**b**), and lead (**c**) at 40 °C (yellow) and 19 °C (blue). Results are displayed as the responses relative to the response at time zero. The dashed lines present ordinary least squares regression fitting results

The short-term stability (STS) was assessed using an isochronous design. The selected bottles were stored at 19 °C and 40 °C to simulate possible transport conditions. The reference temperature was 4 °C. Two bottles were sampled at regular time intervals (15, 28, 42, and 59 days) and placed at the reference temperature until the end of the study. At the end, all samples were measured under repeatability conditions with two independent replicates performed for measurement. The results are presented in Fig. 4. As the short-term study was performed using an isochronous experimental design [15], the measurements were performed under repeatability conditions and data is evaluated as instrumental responses relative to that of time zero. All measurements were performed in randomized order to overcome the effects of potential instrumental drifts. The changes are studied using a zero-order kinetic model where the property values are assumed to change constantly in time. The model parameters are drawn from the linear regression model obtained using ordinary least squares (OLS). The calculation of uncertainty for short-term stability ${u}_{\text{STS}}$^OBJ^) was performed using Eq. 3, considering that the regression slopes were not statistically significant ^OBJ OBJ^. As depicted in Fig. 4, the CRM stability at 40 °C and 19 °C does not differ significantly for arsenic and cadmium. A small difference in the slopes can be observed for lead at the two conditions; however, neither of these slopes differs significatively from zero. The data at 40 °C was utilized, as they simulate extreme transport conditions for the CRM. The STS assessment period was relatively long for a short-term study, but this was intentional, considering that the CRM might eventually present longer-than-usual delays in customs.

3 $${u}_{\text{STS}}=s\left({b}_{\text{STS}}\right)\left({t}_{\text{STS}}\right)$$

where $s\left({b}_{\text{STS}}\right)$ is the standard error for the estimated slope of the linear model and $\left({t}_{\text{STS}}\right)$ is the time interval of the short-time stability study.

**Fig. 5:**
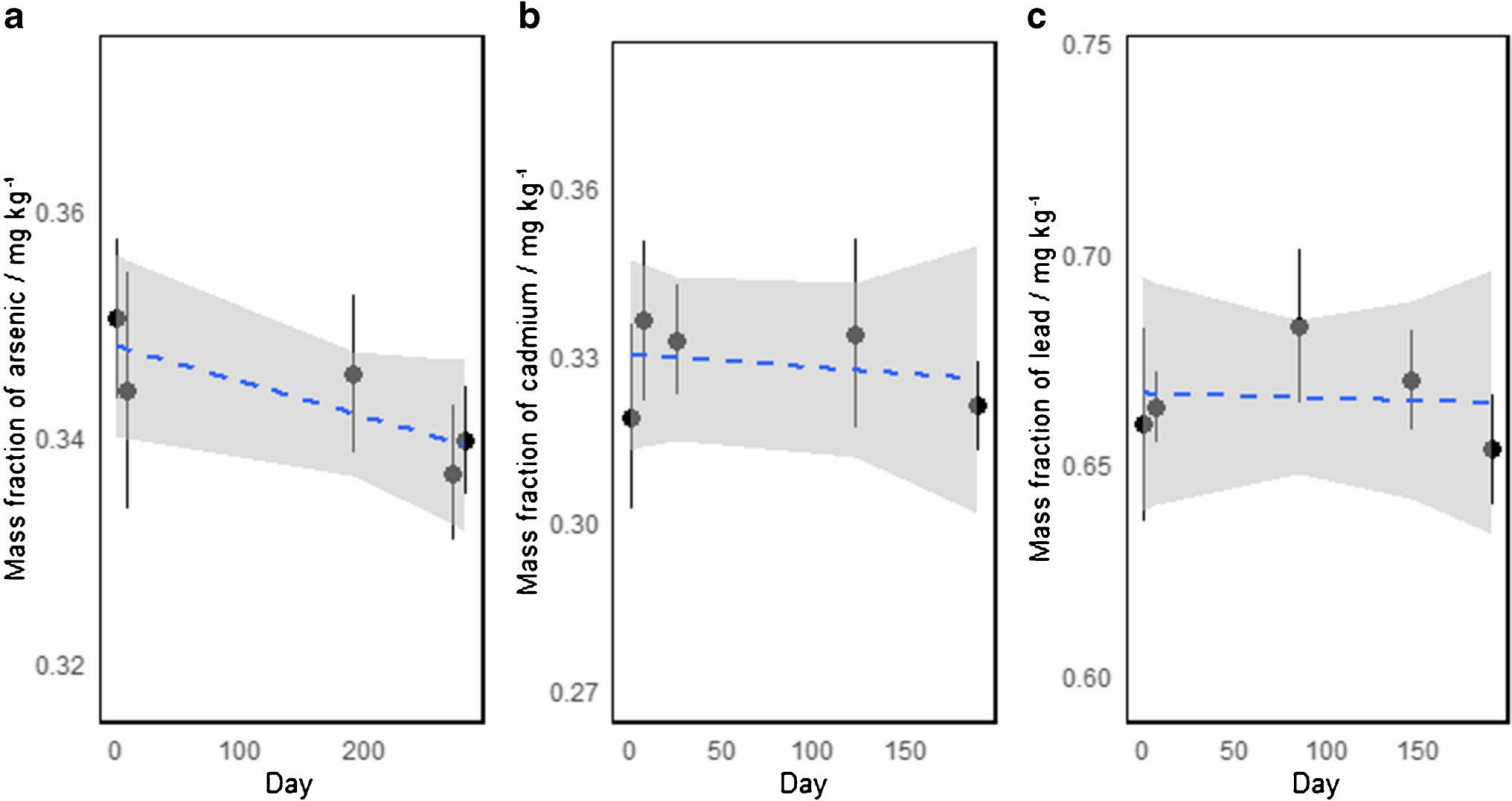
Classical long-term stability study of arsenic (**a**), cadmium (**b**), and lead (**c**) monitored over 6 months. The dashed lines represent the ordinary least square linear fit to model the changes in the property over time and the shaded region represents the 95% confidence interval

The long-term stability (LTS) study was performed using a classical approach, where two bottles were randomly sampled and measured at different time intervals over 6 months [15]. The results are presented in Fig. 5. At each point, the mass fraction of the elements was measured with traceability to the SI. Data treatment was performed using a similar approach as in the STS study, where the data was fitted to a linear function obtained with OLS. The proposed initial validity period of the CRM was 1 year. Equation 4 was used to estimate the LTS uncertainty (${u}_{\text{LTS}}$) of cadmium and lead, as the slopes were not statistically different from zero [15]. On the other hand, the slope for arsenic was different from zero; therefore, the slope instead of the slope error was used for LTS uncertainty calculation. Table 5 presents the results for estimating uncertainty in both short-term and long-term stability studies.

4 $${u}_{\text{LTS}}=s\left({b}_{\text{LTS}}\right)\left({t}_{\text{m}1}+{t}_{\text{cert}}\right)$$

where $s\left({b}_{\text{LTS}}\right)$ is the standard error for the estimated slope of the linear models, ${t}_{\text{m}1}$ is the time interval of the monitoring study and the initial stability monitoring point, and ${t}_{\text{cert}}$ is the estimated lifetime of the CRM after the initial 6-month period.

### Property measurement and value assignment

For the mass fraction of arsenic, cadmium, and lead, two methods of demonstrated accuracy were used: ICP-MS and HG-AAS for arsenic, and ICP-MS and GF-AAS for cadmium and lead. Similar matrix CRMs were used as quality controls in property measurement for the different techniques. The maximal relative error acceptance criterion was that obtained during method validation (5.1%). The results, shown in Table S3 of the supplementary information, yielded relative errors ranging from −3.9 to 2.6%. The combination of measurement results follows the methodology proposed by Levenson [24], which uses the arithmetic mean to average the measurement results, assigns an uncertainty to the average result (${u}_{\text{measur}.}$), and combines the uncertainty with the uncertainty for possible between-methods bias (${u}_{\text{bias}}$) to get the characterization uncertainty (${u}_{\text{char}}$) of the CRM (Eqs. 5–7). The results of combining the measurement results are presented in Table 4.

5 $${u}_{\text{measur}.}=\sqrt{{\left(\frac{1}{2}\right)}^{2}{{u}^{2}(x}_{1})+{\left(\frac{1}{2}\right)}^{2}{{u}^{2}(x}_{2})}$$

6 $${u}_{\text{bias}}=\frac{\left|{x}_{1}-{x}_{2}\right|}{2\sqrt{3}}$$

7 $${u}_{\text{char}}=\sqrt{({u}_{\text{measur}.}{)}^{2}+({u}_{\text{bias}}{)}^{2}}$$

where ${x}_{i}$ and ${u(x}_{i})$ are the mean result and standard measurement uncertainty from measurement method $i (i\in \left(1, 2\right))$.

**Table 4:** Determined mass fractions of arsenic, cadmium, and lead in the CRM INM-040-1

Element	ICP-MS, mg kg^−1^		GF-AAS ^-^ HG-AAS, mg kg^−1^		Combined result, mg kg^−1^			
	Result	*u*_ICP-MS_	Result	*u*_GF-AAS, HG-AAS_	Mean value	*u*_measur._	*u*_bias_	${u}_{\text{char}}$
As	0.347	0.006	0.341	0.013	0.344	0.007	0.002	0.007
Cd	0.332	0.012	0.339	0.009	0.335	0.007	0.002	0.008
Pb	0.661	0.011	0.650	0.017	0.655	0.010	0.003	0.011

The value assignment of the mass fraction of toxic elements in the CRM-040-1 is presented in Table 5. The combined uncertainty of the certified and the reference values (*u*_comb_) includes all known sources of uncertainty determined during the measurement of the property, the between- and within-bottle homogeneity, and the short- and long-term stability. Expanded uncertainties are presented at a confidence interval of approximately 95% using a coverage factor (*k*) of 1.97. The combined uncertainty incorporates the uncertainty components from the property values measurements, the between-bottle homogeneity, and the short-term and long-term stability of the material. The uncertainties were combined by the square root of their quadratic sum. We consider the short-term and long-term stability components independent of each other, since the short-term stability incorporates an additional temperature stability that the CRM might experience during shipping.

**Table 5:** Value assignment and uncertainty budget for CRM of arsenic, cadmium, and lead in the CRM INM-040-1

Element	Assigned value, mg kg^−1^	*u*_char_	*u*_homo_	*u*_STS_	*u*_LTS_	*u*_comb_	*U*_k=1.97_
As	0.344	0.007	0.006	0.003	0.011	0.015	0.030
Cd	0.335	0.008	0.016	0.003	0.014	0.023	0.045
Pb	0.655	0.011	0.015	0.012	0.018	0.028	0.055

## Conclusions

A new CRM of toxic elements in cannabis leaves was prepared and characterized at the INM of Colombia to provide metrological support to the laboratories performing quality control in the cannabis industry. The CRM INM-040-1 is certified for the content of cadmium, lead, and arsenic at mass fraction values of (0.335 ± 0.045) mg/kg and (0.655 ± 0.055) mg/kg, and (0.344 ± 0.030) mg/kg, respectively (expanded uncertainties with a coverage factor *k* = 1.97, at a confidence level of approximately 95%). The CRM is intended to support the development and validation of analytical methodologies, and the quality assurance in laboratories serving the cannabis industry. The value assignment of the certified elements was done by two independent measurement methods of demonstrated accuracy. The combination of the measurement results from two methods lowered the measurement uncertainty from the characterization of the CRM. The CRM is sufficiently stable under transport conditions and can be stored at room temperature for 1 year without suffering significative degradation. The preliminary studies of the small batch of cannabis material and the spiking tests using ethanolic solutions allowed to propose processing conditions that improved the homogeneity of the properties of interest in the CRM.

## Supplementary Information

Below is the link to the electronic supplementary material.

Supplementary file1 (PDF 103 KB)

## References

[1] Balneaves LG, Brown A, Green M, Prosk E, Rapin L, Monahan-Ellison M, et al. Canadians’ use of cannabis for therapeutic purposes since legalization of recreational cannabis: a cross-sectional analysis by medical authorization status. BMC Med. 2024. 10.1186/s12916-024-03370-7. 38589855 10.1186/s12916-024-03370-7 PMC11003000

[2] Mick G, Douek P. Clinical benefits and safety of medical cannabis products: a narrative review on natural extracts. Pain Ther. 2024;13:1063–94. 10.1007/s40122-024-00643-0. 39096481 10.1007/s40122-024-00643-0 PMC11393281

[3] Food and Drug Administration, Cannabis and cannabis-derived compounds: quality considerations for clinical research guidance for industry. Silver Spring, MD: FDA 2023. https://www.fda.gov/drugs/guidance-compliance-regulatory-information/guidances-drugs.

[4] Nelson J, Jones C, Heckle S, Anderson L. Determination of heavy metals in a variety of cannabis and cannabis-derived products, First Action 2021.03. J AOAC Int. 2022. 10.1093/jaoacint/qsab173. 34951636 10.1093/jaoacint/qsab173

[5] Sarma ND, Waye A, Elsohly MA, Brown PN, Elzinga S, Johnson HE, et al. Cannabis inflorescence for medical purposes: USP considerations for quality attributes. J Nat Prod. 2020;83:1334–51. 32281793 10.1021/acs.jnatprod.9b01200

[6] Thomas R. The importance of measuring heavy metal contaminants in cannabis and hemp. Analytical Cannabis. 2021.

[7] Mańkowski J, Kołodziej J, Pudełko K, Kozłowski RM. Bast fibres: the role of hemp (Cannabis sativa L.) in remediation of degraded lands. In: Handbook of natural fibres: Volume 2: Processing and applications. Elsevier Inc.; 2020. 10.1016/B978-0-12-818782-1.00011-0.

[8] Plácido DF, Lee CC. Potential of industrial hemp for phytoremediation of heavy metals. Plants. 2022. 10.3390/plants11050595. 35270065 10.3390/plants11050595 PMC8912475

[9] BIPM.Strategic Plan for the BIPM Work Programme (2022). 2022. https://www.bipm.org/documents/20126/76321966/BIPM-Strategic-Plan-2022.pdf. Accessed 24 Oct 2024.

[10] JCGM. International vocabulary of metrology. 3rd ed. 2021.

[11] McRae G, Bates J, Meija J, Melanson J. HEMP-1: certified reference material of dried, ground hemp. Nat Res Council Canada. 2021. 10.4224/crm.2021.hemp-1.

[12] Wilson WB, Barber CA, Johnson ME, Klingsick JR, Mulloor J, Toman B, et al. Characterization of reference material 8210. 2024. https://nvlpubs.nist.gov/nistpubs/SpecialPublications/NIST.SP.260-248.pdf.

[13] MinComercio, Colombia. Estrategia “Soluciones por la reindustrialización”, lista para impulsar desarrollo de sectores de cannabis, cáñamo y astilleros. 2023. https://www.mincit.gov.co/prensa/noticias/industria/estrategia-soluciones-por-la-reindustrializacion. Accessed 01 Oct 2024.

[14] ISO 17034. General requirements for the competence of reference material producers. Geneva: International Organization for Standardization.2016.

[15] ISO Guide 35. Reference materials — guidance for characterization and assessment of homogeneity and stability. Geneva: International Organization for Standardization; 2017.

[16] Ybañez N, Cervera ML, Montoro R. Determination of arsenic in dry ashed seafood products by hydride generation atomic absorption spectrometry and a critical comparative study with platform furnace Zeeman-effect atomic absorption spectrometry and inductively coupled plasma atomic emission spectrometry. Analytica Chimica Acta. 1992. 10.1016/0003-2670(92)85198-f.

[17] ISO 11095. Linear calibration using reference materials. International Organization for Standardization; 1996.

[18] ISO Guide 33. Reference materials — good practice in using reference materials. Geneva: International Organization for Standardization; 2015.

[19] Pilolli R, Lamonaca A, Nitride C, De Angelis E, van Poucke C, Gillard N, et al. In-house validation of an LC–MS method for the multiplexed quantitative determination of total allergenic food in chocolate. Anal Bioanal Chem. 2024. 10.1007/s00216-023-04894-2. 37615691 10.1007/s00216-023-04894-2 PMC10766722

[20] Paula JFR, Froes-Silva RES, Ciminelli VST. Arsenic determination in complex mining residues by ICP OES after ultrasonic extraction. Microchemical Journal. 2012. 10.1016/j.microc.2012.03.019.

[21] García SS. Especiación de arsénico y acumulación de metales en muestras ambientales. Tesis doctoral. Madrid: Universidad Politécnica de Madrid, Escuela Universitaria de Ingeniería Técnica; 2013.

[22] Henke K, Hutchison A. Arsenic chemistry. In: Arsenic: environmental chemistry, health threats, and waste treatment. 1st ed. Hoboken: Wiley; 2009. p. 18. ISBN: 978-0-470-02758-5.

[23] Ellison SLR. Homogeneity studies and ISO Guide 35:2006. Accredit Qual Assur. 2015;20(6):519–28. 10.1007/s00769-015-1162-z.

[24] Levenson M, Banks D, Eberhardt KR, Gill LM, Guthrie WF, Liu HK, et al. An approach to combining results from multiple methods motivated by the ISO GUM. J Res Natl Inst Stand Technol. 2000. 10.6028/jres.105.047. 27551625 10.6028/jres.105.047 PMC4877152

[25] Meija J, Pagliano E, Mester Z. Coordinate swapping in standard addition graphs for analytical chemistry: a simplified path for uncertainty calculation in linear and nonlinear plots. Anal Chem. 2014. 10.1021/ac5014749. 25099135 10.1021/ac5014749

