# Supplementary material for: Preparation and characterization of a certified reference material of toxic elements in cannabis leaves
Source: Anal Bioanal Chem. 2025 Mar 12;417(12):2691–701. doi: 10.1007/s00216-025-05809-z (PMC12003579; doi:10.1007/s00216-025-05809-z)
Supplement: Supplementary file 1 — Supplementary file1 (PDF 103 KB) [file 216_2025_5809_MOESM1_ESM.pdf]

Supplementary information S1 of

Preparation and characterization of a Certified Reference Material of toxic elements in Cannabis leaves

Adriana Rodriguez [1,2], Cristhian Paredes[1] and Elianna Castillo[2,*]

[1] Grupo de Investigación en Metrología Química y Bioanálisis. Instituto Nacional de Metrología de Colombia, Ak. 50 No. 26-55 Int. 2, 111321 Bogotá, Colombia.

[2] Grupo de Estudios para la Remediación y Mitigación de Impactos Negativos al Ambiente. Universidad Nacional de Colombia, Ak. 30 No. 45-3, 111321 Bogotá, Colombia.

* Corresponding author: ecastillo@unal.edu.co

Table S1. Instrumental linear ranges for measurement method based on ICP-MS.

Analyte isotope/internal estándar	p-value of the lack of fit ANOVA	Instrumental linear range / µg kg^{-1}
**As75 / Ge72**	0.53	0.5 - 99.3
**Cd110 / Ge72**	0.28	0.5 - 101.4
**Cd112 / Tl205**	0.20	0.5 - 101.4
**Cd113 / ln115**	0.08	0.5 - 80.8
**Cd114 / ln115**	0.12	2.5 - 20.6
**Pb206 / Tl205**	0.14	0.5 - 100.5
**Pb207/ Tl205**	0.31	2.5 - 100.5
**Pb208/ Tl205**	0.18	0.5 - 60.2

Table S2. Instrumental linear ranges for measurement method based on GF-AAS and HG-AAS

Element	p-value of the lack of fit ANOVA	Instrumental linear range / µg kg^{-1}
**Pb**	0.49	2.9 – 30
**Cd**	0.21	1.5 - 4.5
**As**	0.13	1.0 – 15

Table S3. Relative error of the certified reference materials (CRM) used as quality controls in property value measurements

Element	Technique	MRC	Relative error / %
As	ICP-MS	Green Tea Leaves SRM 3254	0,3
		Green Tea Leaves SRM 3254	2,6
	HG-AAS	Green Tea Leaves SRM 3254	-0,7
		Kelp Powder SRM 3232	-2,6
Cd	ICP-MS	Pine Needles SRM 1575a	-3,9
		Pine Needles SRM 1575a	-2,5
	GF-AAS	Pine Needles SRM 1575a	1,9
		Pine Needles SRM 1575a	-1,2
Pb	ICP-MS	Apple Leaves SRM 1515	-2,6
		Apple Leaves SRM 1515	-0,88
	GF-AAS	Kelp Powder SRM 3232	1,4
		Kelp Powder SRM 3232	-1,4
